# Effect of hot and cold processed hempseed (*Cannabis sativa*) meal on productive performance, egg quality, egg yolk fatty acid and blood biochemical in laying hens at 37 weeks

**DOI:** 10.1016/j.psj.2026.106693

**Published:** 2026-02-24

**Authors:** Ahmed Ali Sheikh Elmi, Shabana Naz, Rifat Ullah Khan, Muhammad Israr, Yusuf Konca

**Affiliations:** a Department of Animal Science, Faculty of Agriculture, Erciyes University, Kayseri, Türkiye; b Department of Zoology, Government College University Faisalabad, Pakistan; c Physiology Lab, College of Veterinary Sciences, Faculty of Animal Husbandry and Veterinary Sciences, The University of Agriculture, Peshawar, Pakistan; d Task Health Care Limited, 45 Forest Rd, Ilford London, Post code: IG6 3HA, UK

**Keywords:** Hempseed meal, Laying hens, Egg quality, Fatty acid profile, Blood biochemistry

## Abstract

This study evaluated the effects of dietary inclusion of cold- and hot-processed hempseed meal (HSM) on performance, egg quality, yolk fatty acid composition, and blood biochemical parameters in laying hens. A total of 150 Super Nick hens (37 weeks old) were allocated to three dietary treatments with five replicates of eight birds each for 16 weeks: control (0 % HSM), 15 % cold-processed HSM (60 °C), and 15 % hot-processed HSM (120 °C). Performance traits, egg production and quality indices, yolk color, fatty acid composition (gas chromatography), and serum biochemical variables were analyzed. Data were subjected to one-way ANOVA after testing assumptions, and differences among means were considered significant at *P* < 0.05. Final body weight and feed intake were unaffected (*P* > 0.05), whereas feed conversion ratio improved in the cold-processed HSM group compared with the control (*P* = 0.03). Both HSM diets increased hen-day egg production (*P* = 0.01) and yolk pigmentation (Roche score and b*; *P* < 0.01). Hot-processed HSM increased eggshell weight, ratio, and thickness (*P* < 0.05), while internal egg quality traits were unchanged (*P* > 0.05). Yolk linoleic (C18:2n6c) and α-linolenic (C18:3n3) acids increased and oleic acid (C18:1n9c) decreased in HSM-fed groups (*P* < 0.05). Serum triglycerides were reduced in hens fed hot-processed HSM (*P* = 0.04), whereas creatinine, AST, and ALT were not affected (*P* > 0.05). In conclusion, dietary inclusion of 15 % hempseed meal improves feed efficiency, egg production, shell quality, and yolk fatty acid enrichment without adverse physiological effects, with processing temperature influencing the magnitude of responses.

## Introduction

Growing interest in sustainable production systems has led to a focus on alternative feeding strategies that utilize plants such as herbs, vegetables, and their by-products (Chand et al., 2018; Ullah et al., 2022; Almahallawi et al., 2024; Hailat et al., 2024; Hafeez et al., 2025). These feed resources can enhance both sustainability and profitability while delivering healthy end products that meet consumer demand and provide environmentally and economically beneficial solutions (Rehamn et al., 2017; Hafeez et al., 2020; Al-Suwailem et al., 2024; Mushtaq et al., 2025).

Hemp (*Cannabis sativa* L.) is an annual plant that belongs to the Cannabaceae family. Hemp is used in many areas including medicine, cellulose, cosmetics, oil industry and animal feed (Sana et al., 2024). Although it is confused with the type of plant used as an illegal drug, many products can be obtained from the type known as industrial type. In fact, it is often considered as the rediscovery of the plant in recent years (Kanbur, 2022). Hempseed is increasingly cultivated worldwide due to its nutritional value and health benefits. It contains approximately 94.36 % dry matter, 19.1 % crude protein, 6.62 % crude ash, and 32.99 % crude fat, with its oil rich in omega-3 fatty acids (17.69 %) (Konca et al., 2019) and providing about 22 MJ/kg of gross energy (Konca et al., 2019). The fat content ranges from 42 to 47 % in dehulled seeds to 25–34 % in unhulled seeds, while hempseed cake contains 20.40 MJ/kg of gross energy, 298.04 g/kg crude protein, 72.6 g/kg crude ash, and 96.94 g/kg ether extract (Stastnik et al., 2020).

Konca et al. (2019) showed that 15 % heat-treated hempseed improved egg quality traits compared with raw hempseed, while both treatments increased yolk ALA and DHA without affecting blood lipid profile or oxidative stability. Together, these findings indicate that hemp products (15–20 % inclusion) can enrich egg fatty acid composition, and that processing method influences the biological response in laying hens. In line with previous findings on the enrichment potential of hemp products, Mierliță (2019) further demonstrated that inclusion of 20 % cold-pressed hempseed cake increased yolk n-3 fatty acids, reduced cholesterol and the n-6:n-3 ratio, and improved oxidative stability, particularly when combined with tomato by-products as natural antioxidants. Furthermore, hempseed also contains a range of anti-nutritional factors (ANFs) including saponins, trypsin inhibitors, cyanogenic glycosides, condensed tannins, and phytic acid, which can impair nutrient utilization and reduce animal performance (Oomah, 2022). These compounds are of particular concern when incorporating hempseed meal (HSM) into poultry diets, as they may interfere with protein digestibility, mineral bioavailability, and overall feed efficiency.

Hempseed is rich in omega-3 polyunsaturated fatty acids (PUFA), and nutritionists recommend the intake of omega-3 PUFA because of health-promoting effects like antilipidemic effects, demonstrating antithrombotic action and promoting growth (De Briyne et al., 2021; Oomah, 2022; Sana et al., 2024). Omega-3 ratio can be improved in poultry products by changing the fatty acids profile through various seeds and oils in the poultry diet (Konca et al., 2019). Therefore, including hempseed in the poultry diet may change egg yolk's fatty acid composition by enhancing the omega-3 PUFA concentration (Raza et al., 2016).

The nutritional composition of HSM, however, can be influenced by its processing method. Cold processing retains a greater proportion of thermolabile nutrients and polyunsaturated fatty acids, whereas heat processing is frequently applied to deactivate or reduce ANFs such as trypsin inhibitors, thereby potentially improving nutrient digestibility and feed efficiency (Occhiuto et al., 2022). Clarifying how these anti-nutritional factors respond to thermal treatment is critical for evaluating HSM as a feed ingredient. Despite these potential advantages, limited research has been conducted on the comparative effects of cold-processed and heat-processed hempseed meal in the diets of laying hens.

From a biological perspective, the inclusion level and processing of HSM are expected to influence several physiological and production outcomes in laying hens (Kasula et al., 2021). Heat-processed HSM, by reducing ANFs, may improve protein digestibility and mineral absorption, which in turn could enhance egg quality indices such as shell strength and yolk color (Raza et al., 2016; Konca et al., 2019). The high omega-3 PUFA content of HSM is hypothesized to alter yolk fatty acid profiles by increasing the proportion of long-chain n-3 fatty acids, thereby improving the nutritional value of eggs (Öztürk et al., 2024). Furthermore, bioactive compounds present in HSM may influence serum blood metabolites (e.g., lipid profile, total protein), reflecting improvements in metabolic health and antioxidant capacity (Kanbur, 2022). Changes in yolk color metrics may arise from carotenoid pigments and fatty acid interactions, while improvements in villus structure and nutrient absorption could indirectly support better production performance.

Therefore, this study was designed to evaluate the effects of dietary inclusion of hempseed meal processed either by cold pressing, a mechanical method that preserves heat-sensitive nutrients, or by heat pressing, a thermal method that can reduce anti-nutritional factors but may alter some sensitive compounds, on feed efficiency, hen-day egg production, eggshell quality, yolk pigmentation, fatty acid composition, and selected serum biochemical parameters in laying hens, with particular emphasis on how processing method influences production performance, lipid metabolism, and egg nutritional value."

## Materials and methods

The study was approved Erciyes University's local ethics committee released the decision of the approval on 12.04.2023 and 20/120.

### Animals and experimental design

A total of 150 commercial Super Nick laying hens, 37 weeks of age, were used in the study. All birds had been vaccinated against infectious diseases during the chick and pullet stages, following the standard vaccination program used in commercial facilities. During the rearing period, they were fed commercial diets formulated for pullets and laying hens. Before the start of the experiment, all hens were weighed and allocated to treatment groups to minimize differences in body weight among treatments. A 14-day adaptation period was implemented using the control diet, during which egg production was recorded and care was taken to ensure no differences in body weight, egg production, or incidence of deformed eggs among the groups. Hens showing reduced egg production during this period were replaced with healthy, actively laying birds from the same farm. The hens were housed in wire cages (42 × 40 × 46 cm), with two birds per cage. Treatment replicates were distributed across different cage locations, and partitions were installed between feeders to prevent birds from consuming feed from adjacent cages. The lighting program provided 16 hours of light (05:00–21:00) and 8 hours of darkness (21:00–05:00). Following the adaptation period, the experimental diets were fed for 16 weeks (112 days). The birds were assigned to three dietary treatments with 5 replicates per treatment and 10 hens per replicate: The experimental diets in meshed form was consisted of a control diet (conventional corn–soybean meal with no hempseed meal), a diet containing 15 % cold-pressed hempseed meal (HSM-C, processed at 60 °C), and a diet containing 15 % hot-pressed hempseed meal (HSM-H, processed at 120 °C). The 15 % inclusion level was selected based on previous work by Konca et al. (2019), who reported positive effects of 15 % hempseed inclusion on egg quality and yolk fatty acid profile in laying hens. Feed and water were provided ad libitum, and nipple drinkers were used to supply water.

### Experimental diets and feeding procedure

The experimental diets were provided in **mash form**. All feed ingredients, excluding hempseed meal, were obtained from a commercial feed company (**Yem Sanayi ve Ticaret A.Ş., Turkey**). The hempseeds were sourced from **local industrial hemp** and processed according to the method of Konca et al. (2019). **Cold-processed hempseed meal (HSM-C)** was produced using a screw press (**Komet Oil Press, IKA-Werke GmbH & Co. KG, Staufen, Germany**) with oil separation at 60 °C, whereas **hot-processed hempseed meal (HSM-H)** was prepared using the same press at 120 °C. The experimental diets were formulated to meet the nutrient requirements of laying hens according to National Research Council (1994). The control diet contained no hempseed meal, while 15 % HSM-C and 15 % HSM-H were included in the second and third diets, respectively. Feed premix additives were thoroughly mixed using a **mechanical mixer (Patterson-Kelly, East Stroudsburg, PA, USA)** to ensure homogeneity. All feeds were stored in plastic-lidded containers in a shaded area, with fresh batches prepared monthly, and feed was provided **ad libitum**. The ingredient and nutrient compositions of the diets are presented in Table 1.

**Table 1:** Experimental feed composition.

Feed materials (%)	Control	HSM-C, 15 %	HSM-H, 15 %
Corn	52.9	48.3	48.3
Sunflower meal (36 % CP)	1.58	1.58	1.58
Soybean meal (46 % CP)	24.51	15.8	15.8
Wheat	8	8	8
Limestone	9.34	9.2	9.2
Vegetable oil	1.58	1.7	1.7
Salt	0.36	0.35	0.35
Vitamin -mineral Premix^1^	0.2	0.2	0.2
Di-calcium phosphate	1.40	1.38	1.38
D-L-Methionine	0.13	0.10	0.10
Hempseed	0	15	15
Total	100	100	100
Chemical composition*	Laboratory analyses		
Dry matter, %	94.21	94.11	94.28
Crude protein, %	17.51	17.61	17.59
Crude fat, %	3.91	3.89	3.95
Crude fiber	2.63	2.67	2.64
Crude ash	18.76	18.91	18.96
Methionine⁎⁎	0.41	0.42	0.42
Lysine⁎⁎	0.87	0.88	0.88
Calcium⁎⁎	3.87	3.86	3.88
Available Phosphorus⁎⁎	0.39	0.39	0.39
Metabolic Energy Kcal ME/kg***	2760	2755	2750

1 Vitamin-mineral premix per kilogram of the diet, retinol acetate, 10.000,0000 IU; cholecalciferol, 2.500.000 IU; dl-α-tocopheryl acetate, 25.000 mg; menadione, 5.0 mg; Thiamine Hydrochloride, 2.000 mg; riboflavin, 5.000 mg; Pyridoxine Hydrochloride, 5.000 mg; Cyanocobalamin 20 mg; nicotinic acid, 40.000 mg; Calcium D-Pantothenate 12.000 mg, biotin, 50 mg; folic acid, 500 mg, Cantaxanthin, 4,000 mg; β-Apo-8-Carotenic Acid 1.000 mg; β-Apo-8-Carotenic acid 1.000 mg; Manganese (MnO), 120.000 mg; Chlorite, 30.000 mg, Carbonate 5.000 mg; Selenium 250 mg; Antioxidant, 10.000 mg.

⁎⁎ Nutrient compositions were calculated based on (National Research Council, 1994) data of feedstuffs nutrient tables.

### Productive performance

**Conventionally measured productive performance parameters were recorded throughout the experiment. The average live weight of hens was measured individually at the beginning and end of the trial using a precision scale (±1** **g; Sartorius BP 310 S, Sartorius AG, Göttingen, Germany), and body weight (BW) change was calculated as the difference between these values.** Feed intake (FI) was determined **at two-week intervals** by subtracting the **residual feed** from the amount offered to each **replicate**, with **corrections made for mortality to ensure accurate estimates**. The average daily FI per hen was calculated by dividing total feed consumption during each period by the number of feeding days and the number of birds per **replicate**. Feed conversion ratio (FCR) was calculated as the ratio of feed intake to egg mass **for the corresponding period** (Al-Suwailem et al., 2026).

### Determination of egg quality parameters

At the end of the 112-day feeding trial, productive performance and egg quality parameters were determined (Hafeez et al., 2024; Wahyuni et al., 2025). Hen-day egg production was calculated as the number of eggs produced per day divided by the number of live hens on that day, multiplied by 100. For egg quality assessment, five eggs per replicate were collected weekly and the values averaged over the entire period. Egg weight was measured to the nearest 0.01 g using a digital balance, and egg mass was calculated by multiplying the average egg weight by hen-day egg production and dividing by 100. Albumen height was measured using a tripod micrometer on the thick albumen portion of freshly laid eggs. The Roche color score for yolk pigmentation was determined visually using the Roche Yolk Color Fan. Haugh units were calculated from egg weight and albumen height using the standard formula (Kumalasari et al., 2025):

$$\text{HU}=100\;\times \text{lo}g_{10}\left(H-1.7W^{0.37}+7.6\right)$$

where H is albumen height (mm) and W is egg weight (g).

Eggshell weight (g) was recorded after air-drying the shells at room temperature for 24 h. Eggshell ratio (%) was calculated as (eggshell weight / total egg weight) × 100. Eggshell thickness (µm) was measured at three locations (air cell, equator, and sharp end) using a micrometer, and the mean value was recorded. Eggshell surface area (cm²) was estimated using the formula:

$$\text{ESSA}=4.67\;\times W^{0.667}$$

where W is egg weight (g).

Yolk color parameters L* (lightness), a* (redness), and b* (yellowness) were determined on fresh yolk samples using a portable colorimeter (CIELAB system).

### Determination of egg yolk fatty acid composition

**Egg yolk fatty acid (FA) profiles were determined using gas chromatography (GC) following lipid extraction and methylation procedures as described by** Raza et al. (2016)**. For each replicate (cage), five eggs were randomly collected bi-weekly throughout the 16-week experimental period. The yolks were carefully separated from the albumen and pooled within each replicate (cage) to obtain one composite sample per replicate for FA analysis. Thus, the replicate (*n*** **=** **5 per treatment) served as the experimental unit, and fatty acid analysis was conducted on pooled egg yolk samples rather than individual eggs or whole eggs.**

Total lipids were extracted from yolk samples using the **Folch method** with a chloroform:methanol mixture (2:1, v/v; Sigma-Aldrich, St. Louis, MO, USA). Lipids were then converted to fatty acid methyl esters (FAMEs) using **methanolic potassium hydroxide** (Sigma-Aldrich, St. Louis, MO, USA). FAMEs were analyzed using a **gas chromatograph (Agilent 6890N, Agilent Technologies, Santa Clara, CA, USA)** equipped with a **flame ionization detector (FID)** and a **capillary column (SP-2560, 100** **m** **×** **0.25** **mm** **×** **0.20** **μm; Supelco, Bellefonte, PA, USA)**. The injector and detector temperatures were set at 250 °C and 260 °C, respectively. The oven (Agilent Technologies, Santa Clara, CA, USA) was programmed from 140 °C (5 min) to 240 °C at 4 °C/min, held for 15 min. **Helium** was used as the carrier gas at 1.0 mL/min. Individual fatty acids were identified by comparing retention times with a **standard FAME mixture (Supelco 37 Component FAME Mix, Supelco, Bellefonte, PA, USA)**, and results were expressed as **percentage of total identified fatty acids**.

### Determination of blood biochemical parameters

At the end of the feeding trial, 3 mL of blood was collected from the wing vein of three birds per replicate using sterile syringes. The samples were allowed to clot at room temperature for 30 min and then centrifuged at 3,000 rpm for 10 min to separate the serum, which was stored at −20 °C until analysis. Serum biochemical parameters, including triglycerides (TG), creatinine, aspartate aminotransferase (AST), and alanine aminotransferase (ALT), were measured using an automated biochemical analyzer (Roche Cobas C311, Roche Diagnostics, Mannheim, Germany) with commercial diagnostic kits following the manufacturer’s instructions. Specifically, triglycerides were determined using the Triglycerides GPO-PAP kit, creatinine using the Creatinine Jaffe Gen.2 kit, AST using the ASAT (GOT) Gen.2 kit, and ALT using the ALAT (GPT) Gen.2 kit (all from Roche Diagnostics, Mannheim, Germany). All measurements were conducted in duplicate to ensure accuracy and reliability.

### Statistical analysis

All data collected from the experimental groups were first assessed for normality and homogeneity of variance using the Shapiro–Wilk and Levene’s tests, respectively. In cases where data did not meet normality assumptions, appropriate transformations (e.g., log or square-root) were applied. If transformation failed to normalize the data, non-parametric tests such as the Kruskal–Wallis test were employed. Data were analyzed using a **one-way analysis of variance (ANOVA)** because the experimental design consisted of three dietary treatments (control, 15 % cold-pressed HSM, and 15 % hot-pressed HSM). Therefore, the model included **dietary treatment as a fixed effect**. The statistical model used was:

$$Y_{\text{ij}}=\mu \;+\text{Ti}\;+e_{\text{ij}}$$

Where Yij is the observed variable, μ is the overall mean, Ti is the fixed effect of dietary treatment, and e_ij _ is the random error term.

The replicate (cage) was considered the experimental unit for performance, egg quality, fatty acid, and blood parameters. When ANOVA indicated significant differences (*P* < 0.05), means were separated using Tukey’s honestly significant difference (HSD) test. Results are presented as mean ± standard deviation, and statistical significance was declared at *P* < 0.05.

## Results

Table 2 shows that dietary treatment had no effect on final body weight (*p* = 0.355) or average feed intake (*p* = 0.187). Table 2 also indicates that feed conversion ratio (FCR) differed significantly among treatments (*p* = 0.045). Table 2 demonstrates that hens fed cold-processed hempseed meal (HSM-C) had a lower FCR (1.76 ± 0.03) than the control group (1.83 ± 0.02), whereas the HSM-H group (1.77 ± 0.02) did not differ statistically from either the control or HSM-C groups.

**Table 2:** Effects of cold- and hot-processed hempseed meal on final body weight, average feed intake, and feed conversion ratio (FCR, kg feed/kg egg mass) in laying hens.

Parameters	Control	HSM-C	HSM-H	P-value
Final body weight (g)	1650 ± 3.12	1680 ± 5.13	1620 ± 7.13	0.355
Average feed intake (g)	109.74 ± 8.03	108.68 ± 7.91	107.25 ± 6.78	0.187
FCR	1.83 ± 0.02^a^	1.76 ± 0.03^b^	1.77 ± 0.02^ab^	0.045

^ab^ Means within a row bearing different superscripts differ significantly (*P* < 0.05).

**HSM-C:** Cold-processed hempseed meal (15 %).

**HSM-H:** Hot-processed hempseed meal (15 %).

¹Average feed intake represents the mean weekly feed intake per hen averaged over the entire 16-week feeding trial.

150 Super Nick laying hens (37 wk) were randomly assigned to 3 dietary treatments (Control, 15 % HSM-C, 15 % HSM-H), with 5 replicates per treatment and 8 hens per replicate. The 16-week (112 days) trial used the replicate as the experimental unit. Egg production and quality data are means per replicate over the trial.

Table 3 shows that hen-day egg production was significantly affected by diet (*p* = 0.026), with both HSM-C (97.41 ± 0.50 %) and HSM-H (96.79 ± 0.46 %) groups producing more eggs than the control (94.78 ± 0.95 %). Table 3 also indicates that egg weight (*p* = 0.774), egg mass (*p* = 0.088), albumen height (*p* = 0.086), Haugh unit (*p* = 0.310), eggshell surface area (*p* = 0.149), yolk lightness (*p* = 0.095), and yolk redness (*p* = 0.107) were not influenced by treatment. Table 3 further demonstrates that yolk pigmentation increased significantly, as reflected by Roche color score (*p* = 0.001) and yellowness b* value (*p* = 0.001), in both HSM groups compared with the control. Table 3 additionally shows that eggshell weight (*p* = 0.001), eggshell ratio (*p* = 0.001), and eggshell thickness (*p* = 0.002) increased in HSM-H compared to HSM-C.

**Table 3:** Effects of addition of cold and hot processed hempseed meal to diets on egg production and egg quality parameters in laying hens.

	Control	HSM-C	HSM-H	P-value
Hen day egg production (%)	94.78 ± 0.95^b^	97.41 ± 0.50^a^	96.79 ± 0.46^a^	0.026
egg weight (g)	63.22 ± 0.45	63.38 ± 0.53	63.70 ± 0.47	0.774
Egg Mass (g)	60.05 ± 0.75	62.36 ± 0.72	61.39 ± 0.66	0.088
Egg albumin height (mm)	4.50 ± 0.09	4.75 ± 0.08	4.56 ± 0.07	0.086
Roche Color scale	8.67 ± 0.06 ^b^	8.99 ± 0.04 ^a^	8.94 ± 0.04 ^a^	0.001
Haugh unit	88.17 ± 1.09	89.63 ± 0.93	87.67 ± 0.77	0.310
Eggshell weight (g)	5.93 ± 0.04^b^	5.99 ± 0.03 ^b^	6.38 ± 0.03^a^	0.001
eggshell ratio	9.35 ± 0.06^b^	9.38 ± 0.08^b^	9.88 ± 0.06^a^	0.001
Eggshell thickness (µm)	351.1 ± 0.19^ab^	345.7 ± 0.22^b^	356.3 ± 0.20^a^	0.002
Eggshell surface area (cm²)	74.36 ± 0.35	74.75 ± 0.35	75.30 ± 0.31	0.149
Yolk lightness (L*)	56.05 ± 0.20	55.46 ± 0.22	55.55 ± 0.19	0.095
Egg yolk redness (a*)	0.91 ± 0.06	1.26 ± 0.09	1.34 ± 0.24	0.107
Egg yolk yellowness (b*)	36.35 ± 0.33^b^	37.74 ± 0.40^a^	38.53 ± 0.32^a^	0.001

^ab^ Means within a row bearing different superscripts differ significantly (*P* < 0.05).

**HSM-C:** Cold-processed hempseed meal (15 %).

**HSM-H:** Hot-processed hempseed meal (15 %).

150 Super Nick laying hens (37 wk) were randomly assigned to 3 dietary treatments (Control, 15 % HSM-C, 15 % HSM-H), with 5 replicates per treatment and 8 hens per replicate. The 16-week (112 days) trial used the replicate as the experimental unit. Egg production and quality data are means per replicate over the trial.

Table 4 shows that oleic acid (C18:1n9c) decreased in hens fed hempseed meal compared with the control (*p* = 0.038). Table 4 also indicates that linoleic acid (C18:2n6c) (*p* = 0.004) and α-linolenic acid (C18:3n3) (*p* = 0.001) increased significantly in both HSM groups. Table 4 further demonstrates that myristic (*p* = 0.406), palmitic (*p* = 0.665), palmitoleic (*p* = 0.543), stearic (*p* = 0.186), arachidonic (*p* = 0.483), eicosapentaenoic (*p* = 0.757), and docosahexaenoic acids (*p* = 0.193) were not affected by dietary treatment.

**Table 4:** Effects of cold and hot processed hempseed meal to diets on egg yolk fatty acid composition (% of total fatty acids).

Fatty acids	Control	HSM-C	HSM-H	P-value
(C14:0) Myristic Acid	6.23 ± 0.52	6.98 ± 0.42	6.57 ± 0.12	0.406
(C16:0) Palmitic Acid	24.04 ± 5.15	27.90 ± 0.76	26.86 ± 1.14	0.665
(C16:1) Palmitoleic Acid	3.13 ± 0.66	2.57 ± 0.23	2.52 ± 0.21	0.543
(C18:0) Stearic Acid	8.10 ± 0.37	10.07 ± 1.20	8.66 ± 0.10	0.186
(C18:1n9c) Oleic Acid	43.26 ± 0.40^a^	38.35 ± 1.95^b^	40.13 ± 0.53^b^	0.038
(C18:2n6c) Linoleic Acid	14.02 ± 0.45^b^	17.15 ± 0.95^a^	18.76 ± 0.89^a^	0.004
(C18:3n3) a-Linolenic Acid	0.45 ± 0.07^b^	1.10 ± 0.11^a^	1.19 ± 0.14^a^	0.001
(C20:4n6) Arachidonic Acid	0.64 ± 0.05	1.02 ± 0.57	0.87 ± 0.01	0.483
(C20:5n3) Eicosapentaenoic Acid	0.06 ± 0.01	0.07 ± 0.03	0.06 ± 0.01	0.757
(C22:6n3) Docosahexaenoic Acid	0.47 ± 0.01	0.58 ± 0.32	0.38 ± 0.04	0.193

^ab^ Means within a row bearing different superscripts differ significantly (*P* < 0.05).

HSM-C: Cold-processed hempseed meal (15 %).

HSM-H: Hot-processed hempseed meal (15 %).

150 Super Nick laying hens (37 wk) were randomly assigned to 3 dietary treatments (Control, 15 % HSM-C, 15 % HSM-H), with 5 replicates per treatment and 8 hens per replicate. The 16-week (112 days) trial used the replicate as the experimental unit. Egg production and quality data are means per replicate over the trial.

Values are expressed as mean ± SEM (*n* = 5 replicates per treatment). For fatty acid analysis, five eggs per replicate (25 eggs per treatment) were randomly collected during the experimental period and pooled per replicate prior to analysis. The replicate served as the experimental unit for statistical analysis.

Table 5 shows that serum triglyceride concentration differed significantly among treatments (*p* = 0.001), with hens fed hot-processed hempseed meal (HSM-H) exhibiting lower levels (113.29 ± 1.22 mg/dl) than the control (139.29 ± 3.19 mg/dl) and HSM-C (145.43 ± 2.52 mg/dl) groups. Table 5 also indicates that creatinine (*p* = 0.762), AST (*p* = 0.399), and ALT (*p* = 0.432) were not influenced by diet.

**Table 5:** Effects of cold and hot processed hempseed meal to diets on blood biochemical in laying hens.

Groups	Control	HSM-C	HSM-H	P value
Triglyceride (mg/dl)	139.29 ± 3.19^a^	145.43 ± 2.52^a^	113.29 ± 1.22^b^	0.001
Creatinine (mg/dl)	0.21 ± 0.03	0.19 ± 0.02	0.21 ± 0.03	0.762
AST (U/L)	179.14 ± 10.01	169.14 ± 10.17	189.14 ± 10.30	0.399
ALT (U/L)	4.51 ± 0.42	4.32 ± 0.36	4.45 ± 0.11	0.432

Means within a row bearing different superscripts (ᵃᵇ) differ significantly (*P* < 0.05).

HSM-C: 15 % cold-processed hempseed meal.

HSM-H: 15 % hot-processed hempseed meal.

A total of 150 Super Nick laying hens (37 weeks of age) were randomly allocated to three dietary treatments with 5 replicates per treatment and 8 hens per replicate for a 16-week feeding trial.

Values are presented as mean ± SEM (*n* = 5 replicates per treatment). For blood biochemical analysis, **three hens per replicate (10 hens per treatment)** were randomly selected at the end of the 16-week trial, and blood samples were collected individually. The replicate was considered the experimental unit for statistical analysis.

## Discussion

The present study evaluated the effects of dietary inclusion of cold- and hot-processed hempseed meal on **productive performance, egg quality characteristics, yolk fatty acid composition, and selected biochemical indicators** in laying hens. **With respect to conventional performance indicators, final body weight and feed intake were not affected by treatment, indicating that hempseed meal inclusion did not alter maintenance requirements or feeding behavior. While these parameters are reported for completeness, their stability primarily confirms that dietary treatments did not adversely influence general physiological status. More importantly for laying hens,** FCR improved in birds receiving the cold-processed form compared to the control, indicating enhanced efficiency of feed utilization for egg production. **This improvement may be associated with preservation of heat-sensitive nutrients or lipid fractions during cold processing, although this interpretation should be considered a potential explanation rather than a confirmed mechanism** (Öztürk et al., 2024).

Dietary treatments exerted clearer effects on **productive traits**. Hen-day egg production increased in both hempseed-fed groups, supporting previous reports that hemp-derived nutrients and bioactive compounds can positively influence laying performance (Kasula et al., 2021). Despite higher laying rate, egg weight and egg mass remained unchanged, a pattern also reported elsewhere (Öztürk et al., 2024). **This suggests that dietary supplementation may influence laying frequency rather than egg size, reflecting improved reproductive efficiency rather than increased nutrient allocation per egg.**

Eggshell quality parameters responded significantly to dietary hempseed meal inclusion. Hens fed HSM-H exhibited greater shell thickness along with comparable shell weight and shell ratio relative to the cold-processed group, indicating a beneficial effect of heat treatment on shell quality. These improvements may be associated with enhanced nutrient availability resulting from heat processing, which can reduce anti-nutritional factors and improve digestibility of minerals and other key nutrients involved in eggshell formation. Thermal processing may also facilitate structural modifications that enhance calcium utilization and matrix protein deposition. Previous studies have reported that dietary factors influencing mineral bioavailability and nutrient digestibility can significantly impact eggshell quality (Świątkiewicz et al., 2015; Konca et al., 2019; Öztürk et al., 2024). Therefore, heat processing of hempseed meal may improve nutrient accessibility and support better shell matrix integrity, contributing to the observed improvements in shell thickness.

Yolk pigmentation increased markedly in hens fed hempseed meal, as reflected by higher Roche color scores and b* values. This response is consistent with reports that plant-derived pigments contribute to yolk coloration (Öztürk et al., 2024; Rbah et al., 2025). **It is plausible that naturally occurring carotenoids in hempseed meal contributed to this effect, though pigment content was not directly quantified in the present study.** In contrast, albumen height, Haugh unit, and yolk lightness were unaffected, indicating that internal egg quality remained stable and that albumen characteristics were less responsive to dietary modification than yolk attributes.

Dietary hempseed meal markedly altered yolk fatty acid composition. Both processed forms increased linoleic and α-linolenic acids, which is consistent with evidence that dietary fatty acids are efficiently transferred to egg yolk lipids (Silva et al., 2024). The reduction in oleic acid likely reflects proportional redistribution of lipid fractions rather than suppression of its deposition, a phenomenon commonly observed when diets are enriched with polyunsaturated fatty acids (Rbah et al., 2024). Although increases in long-chain n-3 fatty acids such as DHA were not significant, previous studies indicate that conversion of ALA to longer-chain derivatives in hens is limited (Sopian et al., 2025). **Therefore, the observed enrichment primarily reflects direct incorporation of dietary fatty acids rather than metabolic elongation or desaturation.** From a nutritional standpoint, the increased proportion of essential fatty acids may enhance the functional value of eggs for human consumption, **although sensory and storage attributes were not evaluated in this study.**

Among serum biochemical parameters, only triglyceride concentrations were significantly affected, with lower levels observed in hens fed hot-processed hempseed meal. This finding is consistent with reports that dietary polyunsaturated fatty acids can influence lipid metabolism in poultry (Alagawany et al., 2019). The presence of phytosterols in hempseed products has also been associated with lipid-modulating effects (Kanbur, 2022). **It is possible that processing temperature influenced the availability of such bioactive compounds, but this remains speculative because sterol content and digestibility were not measured.** Importantly, creatinine, AST, and ALT were unchanged, indicating no detectable adverse effects on hepatic or renal function, in agreement with previous findings on hempseed supplementation in poultry diets (Konca et al., 2014; Kanbur, 2022).

## Conclusion

Dietary inclusion of hempseed meal—especially in cold-processed form—can enhance feed efficiency, egg production, eggshell quality, and yolk fatty acid composition in laying hens without negatively affecting internal egg quality or blood biochemical. Additionally, hot-processed hempseed meal contributes to lower serum triglyceride levels, supporting its role in promoting healthier lipid metabolism.

## Funding

Not applicable.

## Data availability

The relevant data are provided in the paper. The data of the current experiment can be obtained from corresponding author when needed.

## Ethics approval

The study was approved Erciyes University's local ethics committee released the decision of the approval on 12.04.2023 and 20/120.

## CRediT authorship contribution statement

**Ahmed Ali Sheikh Elmi:** Methodology, Investigation. **Shabana Naz:** Writing – review & editing, Writing – original draft. **Rifat Ullah Khan:** Supervision, Project administration. **Muhammad Israr:** Resources, Funding acquisition. **Yusuf Konca:** Visualization, Validation, Project administration, Methodology, Investigation, Formal analysis, Data curation, Conceptualization.

## Disclosures

No potential conflict of interest was reported by the author(s).
